# Thyroid dysfunction during gestation and gestational diabetes mellitus: a complex relationship

**DOI:** 10.1007/s40618-023-02079-3

**Published:** 2023-04-07

**Authors:** S. Pinto, L. Croce, L. Carlier, E. Cosson, M. Rotondi

**Affiliations:** 1 AP-HP, Department of Endocrinology-Diabetology-Nutrition, Avicenne Hospital, Université Paris 13, Sorbonne Paris Cité, CRNH-IdF, CINFO, Bobigny, France; 2 AP-HP, Ambulatory Unit of Endocrinology-Diabetology-Nutrition, Jean Verdier Hospital, Université Paris 13, Sorbonne Paris Cité, CRNH-IdF, CINFO, Bondy, France; 3 Department of Internal Medicine and Therapeutics, University of Pavia, 27100 Pavia, PV Italy; 4 Unit of Endocrinology and Metabolism, Laboratory for Endocrine Disruptors, Istituti Clinici Scientifici Maugeri IRCCS, Department of Internal Medicine and Therapeutics, University of Pavia, Via S. Maugeri 4, 27100 Pavia, PV Italy; 5 UMR U1153 INSERM/U11125 INRA/CNAM/Université Paris 13, Unité de Recherche Epidémiologique Nutritionnelle, Bobigny, France; 6 NBFC, National Biodiversity Future Center, 90133 Palermo, PA Italy

**Keywords:** Thyroid, Pregnancy, Hypothyroidism, Gestational diabetes mellitus

## Abstract

**Purpose:**

Gestational diabetes mellitus (GDM) and thyroid dysfunction during gestation (GTD) are the two most prevalent endocrinopathies during pregnancy. The aim of the present review is to provide an overview of the peculiar aspects of GDM and GTD, to highlight the potential interactions and clinical consequences of these two frequent clinical conditions.

**Methods:**

A literature review regarding GDM and GTD was carried out with particular interest on meta-analyses and human studies dealing with the (i) shared risk factors between GDM and GTD, (ii) the epidemiological link between GTD and GDM, (iii) physiopathologic link between GTD and GDM, (iv) clinical consequences of GDM and GTD, and (v) post-partum implications of GDM and GTD.

**Results:**

The association between GDM and GTD is common and may be explained by the insulin-resistance state due to maternal GTD, to alterations in the placentation process or to the many shared risk factors. Discrepant results of epidemiologic studies can be explained, at least in part, by the changes in diagnostic criteria and screening strategies throughout the years for both conditions. GDM and GTD impact pregnancy outcome and have post-partum long-term consequences, but more studies are needed to prove an additional adverse effect.

**Conclusions:**

Based on the epidemiological and physio-pathological link between GDM and GTD, it could be suggested that a diagnosis of GTD could lead to screen GDM and the other way round.

## Introduction

During pregnancy, profound hormonal modifications occur, involving both glucose homeostasis and thyroid function adaptation. Several compensatory mechanisms are required to meet the gestation-induced neo-equilibrium [1, 2]. Based on the above, pregnancy may be viewed as a challenging event, which implies that even slight pre-conception reduction in functional reserve (i.e., insulin-resistance or reduced thyroidal reserve) may lead to impaired adaptive responses and subsequent pathological states. This is the case of gestational diabetes mellitus (GDM) and of a wide spectrum of thyroid conditions, including subclinical hypothyroidism (SCH), presence of thyroid autoantibodies in euthyroid women and low maternal FT4 levels, which, for the aim of this review, will be referred to as thyroid dysfunctions during gestation (GTD).

GDM and GTD represent the most prevalent endocrinopathies during pregnancy [3, 4]. Taking into account the diversified screening approach and given the different risk according to ethnicity and body mass index (BMI), the prevalence of GDM ranges between 1 and 28% [5–7]. Reported prevalence for thyroid dysfunction also varies widely across studies according to different diagnostic criteria and included population, going from 0 to 13% for overt hypothyroidism and from 1.5 to 42.9% for SCH [8, 9]. A recent meta-analysis based on the latest American Thyroid Association (ATA) recommendations estimated a prevalence of 0.50% for overt hypothyroidism, 3.47% for SCH, and 2.05% for isolated low maternal fT4 level among pregnant women [10]. The prevalence of thyroid autoantibody positivity also varies widely among studies, with an observed rate among unselected pregnant women ranging from 2 to 17% [11], with a higher rate observed in areas characterized by iodine deficiency [12].

The aim of the present review will be to provide an overview of the relationship between GDM and GTD. The two conditions may indeed share some risk factors for their development, maternal and fetal repercussions as well as long-term consequences. Particular emphasis will be put on the screening strategy of GDM or GTD when the other condition is present.

## Materials and methods

A comprehensive narrative review was performed. We searched for relevant literature using Medline, Embase and Cochrane search and including the following terms: (“gestational diabetes” [MeSH Terms] OR (“gestational diabetes” [All Fields]) AND (“thyroid” [MeSH Terms] OR “thyroid” [All fields]). Publications from 1974 up to 2022 were included.

## Physiopathology of GTD and GDM

During pregnancy, physiologic hormonal changings strongly affect both glucose metabolism and thyroid function. In the first part of gestation an anabolic state establishes in preparation for the energy demands of later pregnancy; this state is characterized by normal or slightly higher insulin sensitivity to promote the glucose uptake by liver and muscle and subsequent reduced fasting glycemia [13, 14]. After this period, human placental lactogen (hPL), human placental growth hormone (hPGH), prolactin and cortisol promote the utilization of glucose by the feto-placental unit, establishing a catabolic status where hepatic gluconeogenesis and insulin resistance progressively increase (≈30% and 50% by late gestation, respectively) [1, 15]. These metabolic adaptations stress β-cells, causing augmented insulin secretion to maintain euglycemia and subsequent β-cell hypertrophy. Pre-pregnancy insulin resistance and/or β-cell dysfunction can trigger a GDM [16].

Pregnancy has also a profound impact on both thyroid gland morphology and function. First, the pre-pregnancy thyroid hormones steady-state equilibrium is markedly modified because of the high circulating levels of human chorionic gonadotropin (hCG) with its thyrotropic action. Moreover, an increase in iodothyronine deiodination activity of the placenta and in the serum concentration of T4-binding globulin occurs [2, 17]. An increase in thyroid volume, due to the stimulation by hCG, is observed throughout gestation. The degree of thyroid volume increase is directly proportional to the degree of iodine deficiency (≈10% in iodine replete countries and ≈20–40% in areas of iodine deficiency) [18]. This increase in thyroid volume is usually reversible at the end of pregnancy [19, 20], but can lead to permanent goiter in areas characterized by iodine deficiency. In the meantime, the production of thyroid hormones increases by nearly 50%, which is paralleled by a ≈50% increase in the daily iodine requirement. These physiological changes are not challenging in thyroid-disease-free women, but different degrees of thyroid dysfunction may occur even in euthyroid women with pre-pregnancy subtle thyroid abnormalities.

Studies performed in non-pregnant women suggest a close link between thyroid function and glucose homeostasis. First, several epidemiologic studies showed that non-pregnant patients with type 2 diabetes (T2D are characterized by a higher prevalence of thyroid dysfunction as compared to the general population [21]. On the other hand, several studies report that, in adult subjects, subtle changes in thyroid hormones levels, even within the normal range, are not free of metabolic repercussions [22–27].

In particular, in the presence of subclinical or overt hypothyroidism, muscle and adipose tissue become resistant to insulin, with a reduced insulin-stimulated glucose uptake in these tissues due to impaired translocation of GLUT4 glucose transporters on the plasma membrane [28, 29].

Given the above, it could be hypothesized that thyroid dysfunction, even if subclinical, could promote a state of insulin resistance also during gestation, and that this phenomenon could contribute to the pathogenesis of GDM during the second part of pregnancy [30].

Another interesting hypothesis regarding the possible physio-pathological link between GTD and GDM stems from the pivotal role of the placenta as a fetal endocrine organ. The placenta has a central role in determining insulin resistance during pregnancy via its secretion of cytokines and hormones, including hCG, hPL, and hPGH, into the maternal circulation [13, 31]. Moreover, the placenta is the main barrier between maternal and fetal environments and regulates the amount of nutrients reaching the fetus.

Indeed, thyroid dysfunction and thyroid autoimmunity in early pregnancy seem to cause an impairment in the development of the feto-placental unit [32]. Recent studies showed that pregnant women with SCH, even in the absence of thyroid autoimmunity, show important abnormalities in surrogate markers of defective placentation, including increased values of the uterine artery pulsatility index and an increased rate uterine artery Doppler velocimetry index abnormalities [33]. Women with thyroid dysfunction also display typical alterations in placenta histological features, including decidual vasculopathy, maternal vascular malperfusion, retroplacental hematoma and subchorionic thrombi [32, 34].

Data coming from histologic analysis of the placenta of women with GDM show a wide array of abnormalities, including placental overgrowth, villous immaturity and edema, vascular abnormalities and altered contractile and vasodilatory responses [35]. Moreover, recent studies suggest that alterations in gene expression and DNA methylation occur in the placental tissue of GDM women [36]. Interestingly, several studies have demonstrated that the placenta is able to secrete circulating microRNAs in maternal blood flow that can influence gene expression in other maternal organs [37, 38].

hCG is a placental hormone that plays a central role in prolonging the progesterone-secreting activity of the corpus luteum and in stimulating trophoblast differentiation, uterine angiogenesis and immunotolerance [39]. Low β-hCG levels are considered a marker of impaired development of the feto-placental unit [40, 41]. Furthermore, a higher hCG in early pregnancy is negatively associated with GDM risk [42–44]. Although no study directly investigated this issue, it could be hypothesized that a defective placentation process in the early phases of gestation due to the presence of thyroid dysfunction could impair placental hormone secretion (including the secretion of beta-hCG) and favor insulin resistance and eventually GDM in the second part of pregnancy.

Moreover, it was recently demonstrated that women with positive anti-TPO antibodies (TPO Ab) display an impaired thyroidal response to hCG in terms of reduced increase in circulating thyroid hormones [45]. The resulting lower FT4 levels could induce a state of insulin resistance, favoring the development of GDM. On the other hand, also pre-existing and/or early pregnancy insulin resistance may affect the placentation process, resulting in lower hCG and subsequent reduced thyroid stimulation with reduced thyroid hormones secretion.

## GDM and GTD: common risk factors?

A possible association between GDM and GTD could be sustained by shared risks factors, as illustrated in the following paragraphs.

### Non-modifiable risk factors

#### Genetic background, ethnicity and environmental exposure

A family history of T2D or hypothyroidism is a known risk factor of GDM [46, 47] or GTD [48], respectively. This phenomenon could reflect the combination of genetic susceptibility and shared environmental exposure and/or lifestyle among households.

For GDM, candidate gene studies identified the association of risk variants and polymorphisms for T2D with GDM, thereby confirming the genetic similarity between GDM and T2D [49, 50]. A shared genetic background for thyroid autoimmunity is suggested by studies on monozygotic twins, with an observed 55% concordance rate [51]. Nevertheless, the role of shared incorrect dietary habits among families [52–54], including a high in fat and sugar diet and a reduced iodine intake, could contribute to the observed association.

There is a general consensus about the increased prevalence of GDM in non-Caucasian populations [55] and in specific ethnic groups (i.e., South Asian, Caribbean, and Middle East [46, 56, 57]). The impact of ethnicity on thyroid dysfunction during pregnancy is less univocal. While some studies suggest a different prevalence of GTD among different ethnic groups [58, 59], other studies do not show any difference [60]. Recent evidence suggests that using ethnic-specific reference ranges for thyroid function parameters during pregnancy would allow to correctly identify pregnant patients with a SCH [61, 62]. A further confounding effect could be due to differences in iodine intake among different ethnic groups due to varying dietary habits and access to multivitamin supplementation [63, 64].

Lastly, the role of the exposure to environmental pollutants during pregnancy in increasing the risk of both GDM [65, 66] and GTD [67, 68] among subjects living in the same area cannot be excluded.

#### Age

The prevalence of GDM increases with age [69, 70], and an age above 35 is often considered as a criterion for GDM screening [70–72].

The most recent ATA guidelines regarding GTD suggest that women older than 30 years should be specifically targeted for thyroid dysfunction screening during pregnancy [48]. Indeed, several studies showed an increase in the prevalence of hypothyroidism with age, reaching a prevalence of ≈10% among women older than 55 years [73, 74]. Although data in the general population suggest that hypothyroidism should be more frequent in older pregnant women, a cohort study specifically designed to evaluate this hypothesis failed to show any increase in the prevalence of hypothyroidism in women older than 30 years when compared with younger women [75].

### Modifiable risk factors

#### Body mass index

It is widely recognized that obesity is a risk factor for both T2D and GDM [70, 76]. On the other hand, to our knowledge, no specific study evaluated if obesity is a risk factor for hypothyroidism during pregnancy. Although this relationship could be hypothesized, literature data coming from the non-pregnant general population suggest that this topic is more complex than what is commonly observed in GDM [77]. Indeed, some population studies seem to show a relationship between BMI and thyroid function [22, 78]. Many authors instead suggest that subjects living with obesity, especially morbid, experience an increase in TSH levels that probably does not reflect a hypothyroid state, but rather a compensatory “hypometabolic” mechanism due to the high fat mass, that quickly recedes after weight loss [77]. A possible role of leptin signaling was suggested to explain this phenomenon [79]. Similarly, while some authors suggest that obesity could be a risk factor for thyroid autoimmunity [80], other showed that the rate of thyroid antibodies positivity was comparable between hypothyroid subjects living with obesity and normo-weight euthyroid controls. Moreover, the male/female ratio commonly found in patients with hypothyroidism was not observed among subjects living with obesity and with higher levels of TSH but no thyroid antibodies [77].

Specific studies in the pregnant population are needed to assess whether obesity has any impact on the risk of hypothyroidism or thyroid autoimmunity during pregnancy, although the case finding ATA screening strategy recommends to test for serum TSH if the pregnant woman has a BMI ≥ 40 kg/m^2^.

#### Vitamin D deficiency

The association between GDM and vitamin D deficiency is still controversial. A recent meta-analysis on pregnant women suggests that vitamin D deficiency could increase the risk of GDM, but the included studies were highly heterogeneous [81]. Some studies suggest that vitamin D supplementation could help reducing fasting plasma glucose and improve insulin resistance in pregnant women [82, 83], although a recent meta-analysis highlighted how the available randomized controlled trials (RCTs) are all of insufficient quality [84]. In conclusion, available evidence is not sufficient to recommend a vitamin D supplementation in GDM-complicated pregnancy to ameliorate the metabolic state and more high-quality RCTs are needed.

Similarly, the association between vitamin D deficiency and hypothyroidism or thyroid autoimmunity during pregnancy is not completely elucidated. Although several studies suggest that non-pregnant subjects with autoimmune thyroid disease have lower serum vitamin D levels as compared to healthy controls [85], only few retrospective studies assessed the correlation between thyroid function and vitamin D deficiency during pregnancy. A recent cohort study, involving 277 pregnant women at 13–28 gestational week (GW), showed that vitamin D levels were positively correlated with TSH, but negatively correlated with free thyroid hormones [86]. On the contrary, other retrospective studies found no association between vitamin D levels and thyroid function parameters throughout gestation [87, 88]. Another study showed a direct linear association between vitamin D serum levels and circulating thyroid hormones, although the association was not seen in the second and third trimester [89]. The postulated mechanism underlying the possible association between thyroid function and Vitamin D levels could be due to the presence of 1,25(OH)D receptors on anterior pituitary [90] and thyroid cells [91], but also to its well-known immune-modulating effect [92, 93]. Nevertheless, the lack of data coming from RCTs does not allow to draw firm conclusions regarding the effects of vitamin D supplementation during pregnancy on thyroid function.

#### Selenium

Selenium has antioxidant properties and is involved in immune function [94]. During pregnancy, there is an increased requirement for selenium due to fetal growth which induces a decrease in maternal blood levels.

Some evidence exists suggesting that selenium levels may be significantly lower in women with GDM than in those without, but the negative correlation has been found only with glycemia and not with insulin levels [95, 96].

Interventional studies in women with GDM gave controversial results: in one RCT the supplementation of 200 μg/d selenium for 6 weeks from 24 to 28 GW was able to reduce fasting plasma glucose, insulin levels and Homeostasis model assessment—insulin resistance (HOMA-IR) [97]. Another RCT showed that a supplementation of 50 µg/d of selenium for 12 weeks starting from the second trimester of pregnancy could reduce fasting plasma glucose [98]. On the contrary, another trial in which pregnant women received 100 µg/d of selenium no effect on fasting plasma glucose, 2-h post-prandial blood glucose, HbA1C, insulin level, and HOMA-IR could be observed [99]. In conclusion, available evidence is not sufficient to recommend a selenium supplementation in GDM-complicated pregnancies to ameliorate the metabolic state and more RCTs are needed.

The thyroid is one of the organs with the highest selenium content because it expresses several specific selenoproteins, including glutathione peroxidases, thioredoxin reductases, iodothyronine deiodinases (responsible for the activation and degradation of T3), and selenoprotein P. The synthesis of thyroid hormones needs adequate levels of selenium [100–102]. Although the role of selenium supplementation as a preventive strategy for post-partum thyroid dysfunction was suggested by a clinical trial [103], no data support the role of selenium deficiency in the pathogenesis of GTD during pregnancy.

#### Myo-inositol

Inositols have insulin-mimetic properties and are able to lower post-prandial blood glucose, delaying carbohydrate digestion and absorption. Furthermore, they can modulate insulin sensitivity [104].

Myo-inositol is one of the predominant forms under which inositols can be found in nature and that have shown to have therapeutic effects in human health; the dysregulation of myo-inositol metabolism is associated with insulin resistance and long-term microvascular complication of diabetes [105]. Because of reduced intake, increased catabolism and excretion, decreased biosynthesis and inhibition of intestinal absorption of myo-inositol, the need of this nutrient is increased during pregnancy [106, 107]. A RCT conducted in women with GDM, comparing the effects of 8-week treatment of different dosage and combinations of inositol stereoisomers on insulin resistance, showed a significant amelioration in HOMA-IR and a lower variation in average weight gain in the treated group [108]. Although some RCTs suggested that myo-inositol supplementation could reduce the incidence of GDM in high-risk women [109–113], a Cochrane review in 2016 concluded that the evidence sustaining the role of myo-inositol supplementation in reducing insulin requirements, reducing plasma glucose and preventing adverse neonatal and pregnancy outcome is still insufficient [114]. A possible explanation for these inconclusive results could be the lack of a universally accepted definition of myo-inositol deficiency, making difficult for the clinicians to specifically target myo-inositol deficient women. Based on the hypothesis that myo-inositol supplementation could limit the need for insulin therapy in women with GDM [115], a double-blind randomized study is ongoing (ClinicalTrials.gov Identifier: NCT03875755).

Myo-inositol is a precursor of inositol triphosphate which plays an important role in TSH in thyroid cell [116]. The effects of myo-inositol supplementation on thyroid function are also not univocal and there is no study evaluating a possible role of myo-inositol supplementation on the incidence of hypothyroidism in pregnancy. Some intervention studies performed in adult non-pregnant subjects showed that a myo-inositol (in association with selenium) supplementation was able to decrease TSH and anti-thyroid antibodies titers in patients with chronic autoimmune thyroiditis [117]. Nevertheless, these results should be confirmed by larger RCTs [116, 118].

#### Cigarette smoking

Available evidence does not suggest that the cigarette smoking during pregnancy is a risk factor for GDM [119, 120].

Some studies performed on non-pregnant subjects suggest that cigarette smoking could reduce TSH, increase thyroid hormones and reduce the probability of developing positivity for anti-thyroid antibodies [121, 122]. Nevertheless, data on pregnant women are few and controversial. Some studies suggest that women smoking during pregnancy display higher levels of FT3, while data on thyroid autoantibody positivity and TSH levels are discrepant among studies [123–125].

## Is GTD a risk factor of GDM?

### The problem of the diagnosis

Answering to the question if GTD is a risk factor of GDM presents several critical issues. Specifically, diagnostic criteria and screening strategies for both conditions may vary.

#### Diagnostic criteria

First, the diagnostic criteria for both GDM and GTD have changed during the last decades.

Diagnostic cutoff of GDM changed over time [126]. The commonly accepted diagnostic criteria for GDM have been defined only in 2010 by the International Association of Diabetes in Pregnancy Study Groups (IADPSG) [127], and then endorsed by the WHO in 2013 [128].

According to IADSPG recommendation, GDM was defined as any between fasting glycemia ≥ 5.2 mmol/l, 1 h OGTT glycemia ≥ 10 mmol/l or 2 h OGTT glycemia ≥ 8.5 mmol/l [127]. This definition encompassed the following sub-types: (i) early-diagnosed GDM, when screening is performed before 24 GW and fasting glycemia is 5.2–6.9 mmol/l, (ii) early diabetes in pregnancy, when before 24 GW fasting glycemia is ≥ 7 mmol/l, (iii) classical GDM, when OGTT is performed after 24 GW and fasting glycemia is 5.2–6.9 mmol/l, 1 h OGTT glycemia ≥ 10 mmol/l or 2 h OGTT glycemia is 8.5–11 mmol/l, and (iv) diabetes in pregnancy, when OGTT is performed after 24 GW and fasting glycemia is ≥ 7 mmol/l or 2 h OGTT glycemia is ≥ 11.1 mmol/l.

The most recent ADA guidelines recommend to define the so-called “early abnormal glucose metabolism” as a fasting glucose above 6.1 mmol/l or an HbA1c above 39 mmol/mol before 24 GW [129]. This change in the diagnostic criteria stems from the fact that the IADPSG diagnostic thresholds for GDM for the 75-g OGTT were not derived from data in the first half of pregnancy. These new cut offs may identify women at higher risk of adverse pregnancy and neonatal outcomes, need for insulin treatment, and post-24 GW GDM diagnosis.

It is evident how the definition of GDM includes a wide spectrum of clinical conditions, including several dysglycemic states during pregnancy according to gestational age and glucose values.

Similarly to what happened for GDM, also the definition of the most frequently observed form of GTD, i.e., SCH, has changed several times in the last decade. According to the most recent ATA Guidelines on Thyroid disease in pregnancy, in the absence of in-house established reference ranges for TSH, a reduction in the upper reference range by ≈ 0.5 mIU/l (corresponding in most cases to a value ≈ 4.0 mIU/l for most TSH assays) should be used to diagnose SCH in a pregnant patient during the first trimester, with a gradual return to pre-pregnancy reference ranges during the second and third trimesters [48]. This novel TSH cutoff actually raised the previous ones, i.e., 2.5 mIU/l during the first and 3.0 during the second and third trimesters. As a result of the increased TSH threshold for diagnosing SCH in pregnancy, a significant decrease in the prevalence of SCH was observed.

#### Screening strategies

Even after reaching an international agreement on the diagnostic criteria for diagnosis of GDM, the recommended screening strategy (universal vs selective, one vs two-step) still differs among different national guidelines [7, 70, 130]. Even when a selective screening is recommended, there is no agreement about which criteria should be followed to select high-risk patients. Some factors (BMI ≥ 25 kg/m^2^, advanced age, non-white ancestry, family history of T2D, previous history of GDM or macrosomia) are widely known to predict the development of GDM [69]. Others, such as parity, male fetus, multiple pregnancy, gestational weight gain, genetic factors, polycystic ovary syndrome, psychosocial factors (for example, depression in pregnancy), unhealthy dietary factors before pregnancy, physically inactive lifestyle before and during pregnancy, steroid or antipsychotic treatment and pregnancy following assisted reproductive technology are not routinely considered when a selective screening is chosen [131].

Also in the case of GTD, there is still no consensus regarding the screening strategy to be employed. Most guidelines recommend testing thyroid function only in women at increased risk, known as case finding, rather than universal screening [48]. According to the most recent ATA guidelines [48], TSH should be tested in case of: a history of hypothyroidism/hyperthyroidism or current symptoms/signs of thyroid dysfunction; known thyroid autoantibody positivity or presence of a goiter; history of head or neck radiation or prior thyroid surgery; age > 30 years, concomitant autoimmune disease; history of pregnancy loss, preterm delivery, or infertility, multiple prior pregnancies (≥ 2); family history of autoimmune thyroid disease or thyroid dysfunction; morbid obesity (BMI ≥ 40 kg/m^2^); use of amiodarone or lithium, or recent administration of iodinated radiologic contrast; residing in an area of known moderate to severe iodine insufficiency.

The case-finding approach overlooks a large number of women with abnormal thyroid function tests [132–136]. However, in a randomized trial, universal thyroid function screening and treatment did not improve overall pregnancy outcomes as compared with testing only high-risk women [137]. Similar findings were observed by a recent case control study, showing that although a case-finding approach may overlook some women with GTD, it is not associated with a higher risk of adverse pregnancy outcomes [138].

### Is SCH associated to an increased risk of GDM?

Several studies evaluated whether the presence of SCH during pregnancy was predictive of development of GDM, as summarized in Table 1. Some studies showed a higher prevalence of SCH among women with GDM when compared with controls [139–143], while other studies [144–148] did not find this association. The varying results of these studies should be interpreted in light of differences in study design, different criteria for diagnosing SCH and GDM during pregnancy and timing of evaluation.

**Table 1:** Clinical studies regarding the relationship between GDM and GTD

Study	Included patients and study design	GDM diagnosis	Evaluation of thyroid function parameters and/or autoimmunity	Thresholds for thyroid function	Evaluation of anti-thyroid antibodies and threshold	Association with GDM	Association with metabolic parameters
Fernández Alba et al. J Clin Med [142]	6775 Pregnant women, retrospective cohort study	Selective (history of familial diabetes in first-degree relatives, BMI > 30 kg/m^2^, history of impaired glucose tolerance or GDM, unfavorable obstetric history, high-risk ethnicity) in the first antenatal visit. Universal screening with two-step at 24–26 GW	TSH, FT4, AbTg, AbTPO at first trimester	Normal TSH and FT4: in-house 2.5th–97.5th percentile (TSH 0.13-4.72 mIU/l, FT4 0.84–1.65 ng/dl); SCH: TSH > 97.5th percentile—10 mUI/l and normal FT4	Yes; AbTPO ≥ 34 IU/l = positive AbTg ≥ 115 IU/l = positive	Increased risk of GDM in women with SCH or with TSH 2.5–4.71 mIU/l or with positive AbTPO	
Chen et al. BMC Endocr Disord [151]	2849 Pregnant women, retrospective cohort study	Universal one-step 24–28 GW	TSH, FT4, AbTg, AbTPO at first trimester	Normal TSH and FT4 in-house 2.5th–97.5th percentile; SCH: TSH > 97.5th percentile and normal FT4	Yes; AbTPO > 34 IU/l = positive	Higher FT4 associated with a lower risk of GDM. No association between TSH, thyroid function sub-types, TPOAb positivity, and GDM risk	
Wang et al. J Clin Endocrinol Metab [158]	6068 Pregnant women, prospective cohort study	Universal one-step	TSH, FT4, FT3, AbTg, AbTPO and FT3/FT4 ratio at first trimester	Normal: FT3 2.20–4.20 ng/dl, FT4 0.80–1.70 ng/dl, TSH 0.30–3.60 mlU/l;	Yes; AbTPO ≥ 100 U/l = positive; AbTg ≥ 70 ng/mL = positive	Higher FT4 associated with a decreased risk of GDM; higher FT3/FT4 ratio associated with an increased risk of GDM after adjusting for potential confounders. No association with Ab titers	FT3 positively associated with fasting and 1-h OGTT, FT4 negatively and the FT3/FT4 ratio positively associated with all OGTT plasma glucose levels
Liu et al. Thyroid [42]	18,683 Pregnant women, prospective cohort study	Universal one-step 24–28 GW	TSH, FT4, FT3, abTg, abTPO and FT3/FT4 ratio before 15 GW	None	Yes; AbTPO > 5.6 IU/ml = positive	Negative association between hCG in early pregnancy and GDM risk. Mediation analysis estimates that 21.4% of hCG-associated GDM risk is mediated through changes in FT4. Negative association between hCG and glycemia during OGTT. In the sensitivity analysis restricted to TPOAb-positive women, no association between hCG and GDM	No association between hCG and fasting glycemia and HbA1c
Safian et al. Curr Diabetes Rev [140]	504 Pregnant women (252 with GDM), retrospective cohort study	At 24–28 GW one-step	TSH, FT4, FT3, AbTPO at 24–28 weeks	Normal ranges: for TSH first trimester 0.1–2.5 mUI/l , second trimester 0.2–3 mUI/l, third semester 0.3–3 mUI/l; for FT4 first trimester 0.26–1.92 ng/dl, second trimester 0.59–1.56 ng/dl, third trimester 0.65–1.26 ng/dl. SCH: TSH between 2.5 and 10 mUI/l and normal FT4. Overt hypothyroidism: TSH ≥ 2.5 mUI/l and low FT4 or TSH ≥ 10 mUI/l	Yes; AbTPO > 100 IU/ml	Higher prevalence of SCH and AbTPO positivity in GDM patients vs controls	
Yang et al. J Clin Endocrinol Metab [157]	27,513 Pregnant women, retrospective cohort study	Universal in the first antenatal visit. Universal one-step 24–28 GW	TSH, FT4 and AbTPO at 8–13 week	None	Yes, NA	Similar prevalence of TPO positivity between GDM and non-GDM, lower TSH and FT4 in GDM, inverse correlation between FT4 and GDM prevalence	
Ying et al. Endocrine [139]	7084 Pregnant women (1141 with GDM), prospective cohort study	Universal first antenatal visit, one-step 24–28 GW	TSH and AbTPO at < 20 weeks	In-house. For the first trimester, normal ranges: TSH 0.06–3.83 mIU/l, FT4 1.01–1.57 ng/dl. For the second trimester, normal: TSH 0.07–4.08 mIU/l , FT4 0.95–1.53 ng/dl	Yes; AbTPO ≥ 60 IU/ml = positive	Prevalence of GDM: patients with normal TSH + negative AbTPOAb 14.65%, high TSH + negative AbTPO 19.57%, normal TSH + positive AbTPO 24.85%, high TSH + positive AbTPO 46.15%	
Haddow et al. PLOS One [148]	9351 Pregnant euthyroid women (272 with GDM). Prospective cohort study	NA	TSH, FT4, FT3, AbTPO at first and second trimester	Normal: TSH between 2nd and 98th centiles at 11–14 GW (0.008–4.17 IU/l), and at 15–18.9 GW (0.05–3.80 IU/l)	Yes; NA	Second trimester FT4 inversely associated to GDM risk, even after adjustment for confounders. No association with FT4 in first trimester	
Knight et al. Eur J Endocrinol [144]	956 Healthy pregnant Caucasian women. Retrospective cohort study	Fasting glycemia, Hba1c, insulin, HOMA-IR at 28 GW; diagnostic criteria NA	TSH, FT4, FT3, abTPO at 28 GW	Manufacturer's population reference. Normal ranges: TSH 0.35–4.5 mIU/l, FT4 11–24 pmol/l, FT3 3.9–6.8 pmol/l. SCH: TSH > 3 mUI/l and normal FT4. Isolated maternal hypothyroxinaemia: FT4 below the 10th centile (< 10.4 pmol/l) with normal TSH	Yes; AbTPO > 34 IU/ml = positive		Maternal FT4 negatively associated with BMI, HbA1c, triglycerides, HOMA-IR but not total/HDL cholesterol ratio. Maternal FT3:FT4 ratio positively associated with BMI, HbA1c, triglycerides, HOMA-IR and total/HDL cholesterol ratio. TSH not associated with any metabolic parameter
Maleki et al. Diabet Med [150]	350 Women with gestational diabetes vs 350 healthy pregnant women. Retrospective case–control study	At 24–28 GW two-step	TSH, FT4 at 24–28 GW	Evaluated at 24–28 GW and post-partum. TSH normal ranges: first trimester, 0.1–2.5 mIU/l; second trimester, 0.2–3.0 mIU/l; third trimester, 0.3–3.0 mIU/l, FT4 normal ranges: first trimester 3.7–23.4 pmol/l (0.26–1.92 ng/dl); second trimester 7.4–18.9 pmol/l (0.59–1.56 ng/dl); third trimester 8.3–15.6 pmol/l (0.65–1.25 ng/dl), post-partum 9.9–28.4 pmol/l (0.77–2.26 ng/dl). Overt hypothyroidism: TSH > 2.5mIU/l and low FT4 or TSH ≥ 10.0 mIU/l. SCH: TSH 2.5–10 mIU/l with a normal FT4	Yes, abTPO > 100 IU/ml = positive; abTg > 150 IU/ml = positive	Higher frequency of thyroid dysfunction in study group (no differentiation between overt and subclinical), higher frequency of post-partum thyroiditis in GDM + thyroid dysfunction	
Karakosta et al. J Clin Endocrinol Metab [149]	1170 Pregnant women. Prospective cohort study	At 24–28 GW 3-h 100-g oral glucose tolerance test with Carpenter and Coustan criteria	Early pregnancy (mean GW 14.1) TSH, FT3, FT4, AbTg, AbTPO	Population-trimester-specific: for the first trimester TSH 0.05–2.53 IU/ml, FT3 2.37–8.02 pmol/l; FT4 12.23–19.69 pmol/l. For the second trimester TSH 0.18–2.73 IU/ml, FT3 2.73–8.13 pmol/l, FT4 11.20–18.66 pmol/l	Yes, AbTPO ≥ 35 IU/ml + positive, AbTg > 40 IU/ml = positive	Patients with high TSH + positive thyroid antibodies in early pregnancy have increased risk for GDM after adjustment for confounders. No isolated effect of antibody positivity	
Vitacolonna et al. Int J Endocrinol [147]	126 Pregnant women (91 with GDM) + 69 (38 with GDM) women who had delivered a baby 18–120 months prior to the investigation. Retrospective cohort study	At 14th–34th GW 3-h 100-g oral glucose tolerance test with Carpenter and Coustan criteria	TSH, FT4, AbTg, AbTPO; GW NA	Kit reference: normal: FT4 0.76–1.42 ng/dl; TH 0.4–4.2 μIU/l	Yes, AbTPO ≥ 15 IU/ml; AbTg ≥ 100 IU/ml	No differences for thyroid function and prevalence of Thyroid Autoantibodies during pregnancy between GDM and controls	
Tudela et al. Obstet Gynecol [143]	24,883 Pregnant women. Retrospective cohort study	Any GW, two-step	1st trimester TSH, FT4	Normal if the 2.5th to 97.5th percentiles, not corrected for gestational age, for the study population (TSH 0.03–4.13 mUI/l, FT4 0.9 to 2.0 ng/dl)	NA	Increased prevalence of GDM with SCH vs euthyroidism, but not after adjustment for confounders	
Montaner et a, Metabolism [145]	619 Pregnant women. Retrospective cohort study	Universal two-step	NA		Yes, AbTPO ≥ 12 IU/ml	No association between AbTPO positivity in early pregnancy and risk of GDM	
Agarwal et al. Gynecol Endocrinol [146]	301 Pregnant women. Prospective cohort study	First trimester fasting glycemia ≥ 95 mg/dl and 2 h post-glucose load ≥ 140 mg/dl were retested by 75 g OGTT in 2 weeks; First trimester fasting glycemia < 95 mg/dl and 2 h post-glucose load < 140 mg/dl were retested by 75 g OGTT with WHO criteria at 24–28 GW	First trimester TSH, FT4, FT3, AbTPO	Population 95% reference normal range: TSH 0.3–4.32 mUI/l; FT4 9.8–18.6 pom/l. Kit normal range for FT3 4.7–802 pmol/l	Yes, AbTPO ≥ 12 IU/ml	No difference in thyroid function or Ab positivity between GDM and controls	
Olivieri et al. Ann Ist Super Sanità [170]	41 Pregnant women at high risk of GDM	Selective (age > 35 y, BMI > 25 kg/m^2^, family history of diabetes, previous macrosomia or GDM and others) 100 g OGTT at 26.9 mean GW	At 26.9 mean GW TSH, FT4, FT3, AbTg, AbTPO	NA	NA	Similar AbTPO and AbTg in high GDM risk and controls	

*GDM* gestational diabetes mellitus, *GTD* gestational thyroid dysfunction, *BMI* body mass index, *OGTT* oral glucose tolerance test, *AbTg* anti-thyroglobulin antibodies, *AbTPO* anti-thyroglobulin antibodies, *TSH* thyrotropic hormone, *FT4* free thyroxine, *FT3* free triiodothyronine, *GW* gestational week, *HOMA* homeostatic model assessment, *IR* insulin resistance, *Ab* antibody, *HbA1c* glycated hemoglobin, *NA* non-available, *SCH* subclinical hypothyroidism

The majority of data come from retrospective cohort studies [139, 142–145, 149], while other studies [140, 147, 150, 151] were designed as case controls (comparing a group of women with GDM and a control of healthy pregnant women). Some authors used a TSH cutoff lower than non-pregnant normal value [143, 147, 150], while other used trimester-specific ranges [139, 142, 144, 149, 151]. In addition, timing of evaluation was not uniform among studies: some studies evaluated thyroid function parameters during the first trimester [139, 142], others during second trimester (usually at the same time of OGTT) [140, 150], or during the third trimester [144, 151].

Keeping these important discrepancies in mind, the main finding that emerges from available studies is that SCH seems to have a predictive role for GDM especially when a higher threshold for defining SCH is considered (TSH > 4.0 mIU/l), particularly in patients with positive thyroid autoantibodies. This finding was confirmed by two recent meta-analyses [152, 153]. The meta-analysis by Kent et al. in particular highlighted how when considering studies using a TSH level < 4.0 mIU/l for SCH diagnosis, no association between SCH and GDM was observed, unless patients were thyroid autoantibody positive. At difference, when a TSH cutoff > 4.0 mIU/l was used, a significant increase in the odds of GDM, regardless of thyroid autoantibody status [153] could be observed. Interestingly, another recent meta-analysis by Maraka et al. failed to show an association between SCH and GDM, but in this case, a TSH value above 2.5 mIU/l was considered, and no correction for the presence of thyroid autoantibodies was performed [154].

A review [155] encompassing several published meta-analyses evaluating risk factors for GDM showed that hypothyroidism (either overt or subclinical) was, surprisingly, the only strongly predictive risk factor for GDM besides obesity, even after correcting for possible biases, although this finding was based on a single meta-analysis including only seven studies [156]. Moreover, the authors were not able to differentiate subclinical from overt hypothyroidism.

The evaluation of the predictive role of TSH as a continuous variable, irrespective from a pre-fixed normality threshold, led to contrasting results. While some studies showed a direct, linear correlation between higher TSH values and GDM risk, even after correction for possible confounders [142, 143, 146], the majority of authors failed to observe a direct correlation between TSH values and GDM risk [42, 144, 148, 151, 157, 158]. Moreover, no direct correlation between TSH levels and metabolic parameters, including, BMI, HbA1c, triglycerides, or HOMA-IR, could be found [144].

In conclusion, the relationship between TSH values and risk of GDM seems to be not linear, with a sharp increase in risk with TSH levels above 4 mU/l and with the additional risk given by the presence of thyroid autoantibodies.

### Can subtle alterations in circulating FT4 increase the risk of GDM, even when TSH is normal?

Several recent studies have highlighted how subtle changes in circulating FT3 and FT4, even with normal TSH, could be linked to an increased incidence of GDM. The most consistently observed finding is a correlation between a combination of low FT4 levels, high FT3 and, consequently, a high FT3/F4 ratio, and a higher risk of developing GDM in late gestation [148, 157–159]. The inverse relationship between FT4 levels and risk of GDM was also confirmed by a recent meta-analysis [160]. Some studies show that FT3 and FT3/FT4 ratio are positively associated with higher fasting glucose levels among women with GDM [159, 161]. Even among healthy pregnant women without GDM a lower FT4 and a higher FT3/FT4 ratio could be related to a worse metabolic profile (i.e., higher BMI, post-OGTT glucose, HbA1c, fasting insulin, HOMA-IR, triglycerides and placental weight) [144, 162].

The physiopathologic reasons underlying of this relationship are not fully understood. One of the possible explanations comes from the role of deiodinases in determining active thyroid hormone availability. T4 is the prohormone of T3 and exerts its effect mainly by converting to T3 through the action of several deiodinase enzymes [163]. For this reason, low levels of FT4 (the metabolically inactive prohormone) and a high FT3/FT4 ratio can be considered as markers for increased deiodinase activity. Obesity has been associated with low FT4 levels [161, 164–166], although through mechanisms that are not completely understood. An increased body weight can increase deiodinase activity, possibly through the action of leptin, resulting in lower FT4 and higher FT3/FT4 ratio [161, 167]. This effect would be even more marked in areas characterized by iodine deficiency [168]. This hypothesis is supported by a recent study demonstrating that a higher expression and activity of Type 3 Deiodinase can be observed in the placental tissue of mothers with GDM [141]. According to this hypothesis, alterations in circulating thyroid hormones would be a consequence, rather than a cause, of the higher BMI typical of women with GDM.

Another interesting hypothesis was suggested by a recent study including more than 18,000 women in China aimed at identifying if there was a relationship between hCG levels in early pregnancy and the risk of GDM, and if this was mediated by FT4 levels. The results showed that higher hCG in early pregnancy were associated with a lower GDM risk in TPO Ab negative women, but not in TPO Ab positive ones. The authors hypothesized that lower hCG in early pregnancy and/or the presence of TPO Ab could lead to alterations in the placentation process and reduced hCG-mediated thyroid stimulation with a subsequent lowering in FT4 levels, both leading to an increased risk of insulin resistance and GDM [42].

### Is thyroid autoantibody positivity a risk factor for GDM?

Few and weak evidences supported an independent association of thyroid autoantibodies positivity and GDM [169]. An early report by Olivieri et al. suggested that women with multiple risk factors of GDM were more frequently positive for thyroid autoantibodies (16% vs 11.7%), independently from thyroid function [170]. No difference in thyroid autoantibodies prevalence was instead observed in GDM vs healthy pregnant women in several more recent studies [145, 157, 171, 172]. Moreover, Karakosta et al. [149] showed that the sole presence of thyroid autoantibodies in euthyroid women did not confer an increased risk of GDM.

A meta-analysis published in 2015 specifically aimed at assessing the link between GDM and the presence of thyroid autoantibodies, showed only a weak association between thyroid autoantibodies and GDM. The sub-group meta-analysis highlighted that a significant positive association could be found in women with concurrent thyroid dysfunction, but not in euthyroid women [173].

In conclusion, available literature suggests that an association between thyroid autoantibodies positivity and GDM can be observed only when a thyroid dysfunction is present.

## Do GDM and GTD impact on the same pregnancy-related outcomes?

The pregnancy complications of the two conditions are summarized in Table 2.

**Table 2:** Maternal and fetal outcomes

	GDM	GTD
Pre-eclampsia	X	X
Preterm delivery (37 GW)	X	X
Large for gestational age	X	
Shoulder dystocia	X	
Neonatal hypoglycemia	X	
Admission to newborn intensive care	X	
Small for gestational age		X
Pregnancy loss		X
Lower offspring IQ		X

*GDM* gestational diabetes mellitus, *GTD* gestational thyroid dysfunction, *IQ* intelligence quotient

The Hyperglycemia and Adverse Pregnancy Outcome (HAPO) Study [174] demonstrated that maternal hyperglycemia increases the risk of adverse pregnancy outcomes for both the newborn (including macrosomia, shoulder dystocia, neonatal hypoglycemia, intensive neonatal care and hyperbilirubinemia) and the mother (including preeclampsia, cesarean section and premature delivery). Indeed, different GDM sub-types and/or increases in different parameters of OGTT (fasting, 1 h or 2 h post-load) may have different prognostic relevance in terms of adverse pregnancy outcomes [127, 175]. Treating GDM can prevent, at least in part, the occurrence of these complications [127, 176, 177].

Evidence about the efficacy of screening and treating early-pregnancy abnormal glucose metabolism is conflicting and this depends on small sample size, different screening strategies with different diagnostic thresholds, heterogeneity in age, BMI and ethnicity of studied populations [178–180]. A recent meta-analysis [181] did not find any difference in several pregnancy outcomes among all the trials comparing early screening and treatment of dysglycemia to routine care, even if a sub-group analysis of trials that performed a universal screening, as opposed to only including GDM high-risk women, demonstrated a lower rate of large-for-gestational-age with early screening and treatment for GDM. Two large RCTs, (TESGO study/Taiwan, NCT 03523143 and LEMA_GDM, NCT04451915) are ongoing to better clarify the matter [182].

Overt maternal hypothyroidism was long known to be associated with an increased risk of adverse pregnancy complications [183] and subsequent impairment of fetal neurocognitive development [184]. More specifically, overtly hypothyroid pregnant women display increased risks of gestational hypertension, premature birth, low birth weight, pregnancy loss, and lower offspring intelligence quotient (IQ) [185, 186]. Based on the above findings, it is clear that overt hypothyroidism negatively affects the maternal–fetal unit.

On the other hand, GTD has been less consistently associated to adverse pregnancy outcomes which, at least in part may depend upon different TSH cutoffs used for defining SCH and by the non-systematic evaluation of thyroid autoantibodies status. In particular, the notion that thyroid autoantibodies positivity per se may affect pregnancy outcome independently from thyroid function has further complicated the issue.

The main end-points for which the role of GTD was evaluated include: effects on pregnancy outcome (i.e., pregnancy loss), adverse perinatal outcomes (i.e., premature delivery and hypertensive disorders), and neurocognitive outcomes in offspring. Although the results of the published studies remain contrasting, according to a meta-analysis evaluating the risk of pregnancy complications (pregnancy loss, preterm delivery, and placental abruption) in relation to maternal thyroid status, a significant association with SCH during early pregnancy would actually be present. It should be highlighted that SCH was variably defined across the included studies [154]. A recent cohort study including more than 8000 pregnant women showed that a maternal TSH above 4 mIU/l was associated with an approximately twofold increased risks of prematurity and respiratory distress syndrome in the offspring [187].

## Post-partum implications

### GDM

#### Mother

After delivery, in most cases, euglycemia is restored, but GDM will recur in 30–84% of subsequent pregnancies, according to ethnicity [188]. Weight gain between pregnancies and post-partum weight are also important risk factors for GDM recurrence [188].

Moreover, a history of GDM confers a sevenfold increased risk of T2D in the mother [188–190], and a post-partum OGTT and life-long metabolic follow-up are recommended to screen a persistent dysglycemia or a new onset of prediabetes or diabetes, respectively [191]. Furthermore, GDM diagnosed by different criteria (IADSPG vs Carpenter&Coustan) was found differently associated to persistent prediabetes or overt diabetes, being IADSPG criteria less predictive [192].

An interesting meta-analysis found that the risk of T2D is increased mostly in case of raised fasting glucose, need of insulin during pregnancy, and mostly in case of early-diagnosed GDM [193]. Moreover, women with a previous GDM display an elevated cardiovascular risk, even in the absence of development of T2D and women with a history of GDM who become diabetic after pregnancy have an increased risk of microvascular complications as compared to T2D women without a previous GDM [194, 195].

#### Offspring

Maternal GDM could affect the metabolic status of offspring all life-long. In fact, a higher risk of obesity and dysglycemia during infancy and adulthood has been shown in the sons and daughters of women with GDM [196–200]. Several studies agree about the role of fetal GDM exposure on the development of neuro-psychiatric disorders (substance use disorders, schizophrenia, mood and anxiety disorders, eating disorders, intellectual and developmental disorders) in childhood and adulthood [201, 202]. It should be emphasized that a part of this effect could be due to preeclampsia, a condition that is known to be associated to increased risk of psychiatric disorders [203].

### GTD

#### Mother

According to a recent study, almost 40% of patients with SCH during pregnancy showed a persistent hypothyroidism after delivery during a median follow-up of 11 months, with anti-thyroid antibody positivity during pregnancy and persistency at 6 weeks after delivery as the significant risk factors for long-term hypothyroidism. Moreover, almost one-third of women with normal thyroid function 6-week post-partum were found to have hypothyroidism during the follow-up [204]. In another study, where women were followed for 20 years, overt hypothyroidism and TPOAb increased the risk for subsequent thyroid disease 17- and 4.2-fold, respectively, showing a synergic effect [30].

It is established that family history of thyroid dysfunction is a risk factor for personal thyroid dysfunction. Genetic background is important, but fetal programming may have a role [205]. Experimental data from animal suggest that deficiencies of thyroid hormones during gestation and lactation can alter the thyroid function and the metabolic status of the fetus [206].

#### Offspring

The effects of maternal SCH on fetal neurocognitive development are by far less clear. Some previous studies indicated a slight but significant reduction in IQ among children as well as a delay in motor skill development, language development, and attention at 7–9 years of age in children born to untreated women with gestational SCH compared to euthyroid controls [184]. Some studies suggest that even the positivity for thyroid autoantibodies, in the absence of SCH, could cause detrimental effects on children neurological development, even if there is great discrepancies among studies [207]. However, intervention studies in pregnant women with SCH receiving placebo or LT4 did not differ for maternal and fetal outcomes. Potential limitations of these negative results were suggested to be related to the delayed initiation of treatment with LT4 (approximately at 13 and 16 weeks of pregnancy, respectively) [208, 209].

### Association of GTD and GDM

Data regarding the long-term consequences of GDM and GTD when the two conditions are associated are still scanty. The presence of overt hypothyroidism during pregnancy seems to confer a sixfold higher risk for developing T2D throughout a 20-year follow-up, even after adjustment for age, BMI, parity and a history of GDM [30]. Moreover, some evidence suggests that post-partum thyroiditis is more frequent in women with a history of GDM than in healthy pregnant women [150].

## Conclusions

GDM and GTD are the most common endocrinopathies found in pregnant women. Several studies suggest that these two conditions often co-occur. This association may be explained by the induced insulin resistance state due to the action of thyroid hormones on the mother. Another possible explanation resides on the alterations in the placentation process which are typical of both GDM and GTD. Furthermore, the association between GDM and GTD could be sustained by several shared risk factors. Lastly, the possible role of GDM as a cause of thyroid dysfunction has not been investigated up to now, but could be the topic of future studies.

Based on the evidence of a possible epidemiological link between GDM and GTD, it could be suggested that a diagnosis of GTD could lead to screen GDM and the other way round.

Furthermore, both conditions are associated with an increased risk of pregnancy complications, most importantly preeclampsia and preterm delivery, so it could be hypothesized that the early management of the two could further improve pregnancy outcomes. Although in the past years, many studies have evaluated the epidemiological correlation between GDM and GTD, data regarding some specific clinical issues are lacking. These include the potential: (i) impact on the therapeutic management of GDM and GTD when the two conditions coexist; (ii) benefit of early treatment of GDM and GTD on pregnancy outcome; (iii) differences in terms of post-partum outcomes (both early and long-term) of GDM and GTD on the mother and the offspring when the two conditions are associated. Further prospective studies on these topics are needed to provide this essential information to the clinical community and to ameliorate the management of these two very common pregnancy-associated conditions. In the meanwhile, since it is highly probable that the coexistence of these two common disorders may result in a worse pregnancy outcome, these pregnant patients should be followed up with particular attention and care.

## Data Availability

Data sharing not applicable to this article as no datasets were generated or analysed during the current study.
